# Characterization, management, and risk factors of hyperglycemia during PI3K or AKT inhibitor treatment

**DOI:** 10.1002/cam4.4579

**Published:** 2022-02-25

**Authors:** Dazhi Liu, Michael A. Weintraub, Christine Garcia, Marcus D. Goncalves, Ann Elizabeth Sisk, Alissa Casas, James J. Harding, Stephen Harnicar, Alexander Drilon, Komal Jhaveri, James H. Flory

**Affiliations:** ^1^ Department of Pharmacy Memorial Sloan Kettering Cancer Center New York New York USA; ^2^ Department of Medicine Memorial Sloan Kettering Cancer Center New York New York USA; ^3^ Department of Medicine Weill Cornell Medical College New York New York USA

**Keywords:** hyperglycemia, PI3K/AKT inhibitors, risk factors, SGLT2 inhibitors, toxicity management

## Abstract

**Purpose:**

The phosphoinositide 3‐kinase (PI3K)/protein kinase B (AKT) pathway controls insulin sensitivity and glucose metabolism. Hyperglycemia is one of the most common on‐target adverse effects (AEs) of PI3K/AKT inhibitors. As several PI3K and AKT inhibitors are approved by the United States Food and Drug Administration or are being studied in clinical trials, characterizing this AE and developing a management strategy is essential.

**Methods:**

Patients with hematologic or solid malignancies treated at Memorial Sloan Kettering Cancer Center with a PI3K or AKT inhibitor were included in this retrospective analysis. A search for patients experiencing hyperglycemia was performed. The frequency, management interventions and outcomes were characterized.

**Results:**

Four hundred and ninety‐one patients with 10 unique cancer types who received a PI3K or AKT inhibitor were included. Twelve percent of patients required a dose interruption, 6% of patients required a dose reduction and 2% of patients were hospitalized to manage hyperglycemia. No events occurred among patients receiving *β*‐, *γ*‐, or *δ*‐ specific PI3K inhibitor. There was one case where the PI3K or AKT inhibitor was permanently discontinued due to hyperglycemia. Metformin was the most commonly used antidiabetic medication, followed by insulin, sodium‐glucose transport protein 2 (SGLT2) inhibitors, and sulfonylurea. SGLT2 inhibitors were associated with the greatest reductions in blood sugar, followed by metformin. At least one case of euglycemic diabetic ketoacidosis (DKA) occurred in a patient on PI3K inhibitor and SGLT2 inhibitor. Body mass index ≥ 25 and HbA_1c_ ≥ 5.7 are were independently significant predictors of developing hyperglycemia.

**Conclusion:**

Hyperglycemia is one of the major on‐target side effects of PI3K and AKT inhibitors. It is manageable with antidiabetic medications, treatment interruption and/or dose modification. We summarize pharmacological interventions that may be considered for PI3K/AKT inhibitor induced hyperglycemia. SGLT2‐inhibitor may be a particularly effective second‐line option after metformin but there is a low risk of euglycemic DKA, which can be deadly. To our knowledge, our report is the largest study of hyperglycemia in patients receiving PI3K/AKT inhibitors.

## Graphical abstract

Phosphoinositide 3‐kinase (PI3K)/protein kinase B (AKT) inhibitor induced hyperglycemia is particularly likely to happen in patients with baseline diabetes, pre‐diabetes, or elevated body mass index. It is manageable with dose interruption, dose modification and/or pharmacologic management; and that sodium‐glucose transport protein 2 inhibitors are a promising second‐line option after metformin.

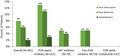

## 1 INTRODUCTION

The PI3K‐AKT pathway plays essential roles in cell differentiation, proliferation and survival.^1^ Isoform specific inhibitors of this pathway are approved by regulatory agencies for the treatment of certain types of lymphoma and breast cancer.^2^, ^3^, ^4^, ^5^, ^6^, ^7^, ^8^ For example, alpelisib is a selective inhibitor against the α‐isoform of PI3K that is effective in postmenopausal women and men with hormone receptor (HR) positive, human epidermal growth factor receptor 2 negative, *PIK3CA* mutated advanced or metastatic breast cancers when used together with fulvestrant.^9^ It leads to statistically longer progression free survival and numerically longer overall survival (OS) compared to fulvestrant only, and is currently approved in the United States, Australia, and Europe.^9^, ^10^ Copanlisib, an inhibitor targeting the α and δ isoforms of PI3K, is approved in the United States for relapsed patients with follicular lymphoma who received at least two prior systemic therapies.^11^, ^12^ Several other PI3K and AKT inhibitors are being studied in cancers with genetic alterations in the PI3K‐AKT pathway.^13^, ^14^, ^15^

The use of PI3K‐AKT inhibitors has been limited by several adverse events (AEs) including hyperglycemia, rash, diarrhea, pneumonitis and colitis.^16^, ^17^, ^18^ Hyperglycemia is the most common AE occurring in up to 80% of subjects on clinical trials.^19^, ^20^, ^21^ Cellular PI3K and AKT activity are critical for controlling glucose homeostasis, and animal studies have shown that p110*α* inhibitors, but not p110*β* or p110*δ*, can block adipocyte insulin‐dependent glucose uptake and regulation.^22^, ^23^ AKT is known to regulate hepatic glycogenolysis and glucose uptake through its substrate, glycogen synthase kinase‐3.^16^ Therefore, hyperglycemia following inhibition of the PI3K‐AKT pathway is an on‐target effect and a clinical management strategy is needed to prevent and mitigate this response. In large phase III trials, metformin was recommended as the first step to improving hyperglycemia, but this approach was effective in only 40% of cases.^19^, ^24^ Currently, there are no clear guidelines on how to manage hyperglycemia when metformin is not effective. Moreover, the risk factors for hyperglycemia are not yet fully understood. With more PI3K and AKT inhibitors being approved or entering clinical trials, further characterizing the hyperglycemia associated with PI3K or AKT inhibition and defining potential risk management strategies is an unfulfilled need. To address this need, we report the frequency, risk factors, management, and outcomes of hyperglycemia secondary to PI3C and AKT inhibitors at a large cancer center from 1 January 2014 to 31 December 2020.

## 2 METHODS

This is a single‐center, retrospective study to identify the rate of treatment interruption/discontinuation due to hyperglycemia in patients treated with PI3K/AKT inhibitors. Data on patients who received at least one dose of a PI3K or AKT inhibitor at Memorial Sloan Kettering Cancer Center between 1 January 2014 and 31 December 2020 were retrospectively analyzed. Demographics, blood glucose, and hyperglycemia management were obtained from electronic records and pharmacy databases, using data through 31 December 2020.

The duration of exposure to PI3K and AKT inhibitors and antidiabetic drugs was identified through electronic prescribing records. Start and stop dates, as well as all dose changes and periods of treatment interruption, were confirmed for all patients through manual chart review. Baseline characteristics including demographics, cancer diagnosis, comorbidities, and serial serum creatinines, estimated glomerular filtration rates, body mass indices (BMIs), and glycated hemoglobins (HbA_1c_) were also extracted from the electronic medical record.

The primary outcome of the study was disruption in treatment attributed to hyperglycemia, which included interruption in treatment, dose reduction, discontinuation of drug, and hospitalization for hyperglycemia. Secondary outcomes included use of antidiabetic drugs (including metformin, SGLT2 inhibitors, dipeptidyl peptidase 4 (DPP4) inhibitors, sulfonylureas, insulin, thiazolidinediones, and glucagon‐like peptide‐1 (GLP1) receptor agonists). Serial random glucoses during treatment were derived from MSKCC laboratory data, graded as hyperglycemia according to the Common Terminology Criteria for Adverse Events version 4.0, and included as a secondary outcome.

All chart reviews were conducted by coauthors DL, CG, MW, and JHF. Twenty percent of charts were reviewed by at least two reviewers to ensure interrater reliability. Cases of ambiguous documentation of treatment dates were resolved by consensus among co‐authors.

The association between pertinent risk factors and the incidence of hyperglycemia was analyzed using univariate logistic regression. Risk factors were chosen a‐priori and included baseline BMI, HbA_1c_, use of antidiabetic medications (metformin, sulfonylurea, SGLT2 inhibitor, GLP1 receptor agonist, DPP4 inhibitor, thiazolidinedione, alpha glucosidase inhibitor, meglitinide, or insulin), mean random blood glucose, age, and sex. Patients were also categorized based on whether they had baseline diabetes, as defined by an HbA_1c_ ≥ 6.5 or use of antidiabetic medications. Univariate odds ratios were calculated by Fisher's exact test performed using GraphPad Prism 7 (GraphPad Software, Inc). R 4.0.0 was used for data analysis. All hypothesis testing was two‐sided with a 5% level of significance (*p* < 0.05).

This study was approved by the MSKCC Institutional Review Board.

## 3 RESULTS

### 3.1 Cohort description

Four hundred and ninety‐one patients were identified. The median age was 62 years (range 20–88 years) and 70% of patients were women (Table 1). Nine unique cancer diagnoses were represented, the most common of which was breast cancer (47%), followed by leukemia/lymphoma (19%), genitourinary cancers (8%), and central nervous system cancers (6%). Most patients received PI3K inhibitors targeting the *α* isoform (51%). One hundred and forty‐two patients received *γ* or *δ* isoform specific PI3K inhibitor (29%), followed by AKT inhibitor (16%) and pan PI3K inhibitor (4%). Seven percent of patients (*n* = 32/491) were on at least one medication for elevated blood glucose prior to PI3K or AKT inhibitor therapy. BMI, HbA_1c_, and random glucose values are also summarized in Table 1.

**TABLE 1:** Demographic. The clinical features of 491 patients with liquid or solid malignancies that were treated with a PI3K or AKT inhibitor are summarized

	*n* = 491 patients
Sex	
Male	30% (149)
Female	70% (342)
Age	
Median (range)	62 years (22–88 years)
0–50	17% (85)
50–65	43% (212)
65 or above	40% (194)
Tumor type	
Breast	47% (232)
Lymphoma/Leukemia	19% (95)
GU	9% (44)
CNS	6% (30)
Gyn	6% (29)
GI	5% (26)
ENT	4% (19)
Lung	2% (11)
Thyroid	1% (2)
Drug	
Alpha isoform inhibitor	51% (251)
Alpelisib	44% (217)
Copanlisib	5% (26)
Others	2% (8)
PI3K β/δ inhibitor	29% (142)
Duvelisib	15% (76)
Idelalisib	10% (47)
Others	4% (19)
AKT inhibitor	16% (79)
Pan PI3K inhibitor	4% (19)
Baseline body mass index	
<25	37% (183)
25–30	39% (192)
≥30	24% (115)
Baseline diabetes medications	
Metformin	5% (25)
Insulin	3% (15)
Sulfonylurea	2% (12)
DPP4 inhibitor	2% (7)
SGLT2 inhibitor	<1% (3)
GLP1	<1% (1)
Baseline A1C	
0.0–5.5	37% (180)
5.6–6.5	17% (84)
>6.5	4% (18)
Unknown	43% (209)
Baseline glucose	
0–100	44% (216)
101–110	27% (132)
111–140	21% (104)
>140	5% (25)
Unknown	3% (14)
Treatment duration (days)	
Alpha isoform inhibitor	81 (42–174)
PI3K β/δ inhibitor	96 (48–198)
AKT inhibitor	109 (56–214)
Pan PI3K inhibitor	51 (33–94)

Abbreviations: AKT, Protein kinase B; DPP4, dipeptidyl peptidase‐4; GLP1, glucagon‐like peptide‐1; PI3K, phosphoinositide 3‐kinases; SGLT2, sodium/glucose cotransporter 2.

### 3.2 Treatment disruption due to hyperglycemia

**FIGURE 1:**
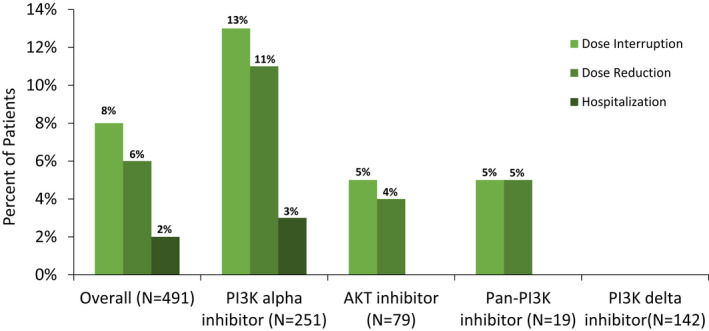
Frequency of interventions. The frequency of dose interruption, dose reduction, and hospital admission to manage hyperglycemia associated with PI3K or AKT inhibitor use are shown. The prevalence of each intervention in each drug class is displayed. AKT, protein kinase B; PI3K, phosphoinositide 3‐kinase

Twelve percent (39/491) of patients required a dose interruption and 6% (30/491) of patients required a dose reduction due to hyperglycemia. All such hyperglycemia‐associated treatment disruptions occurred in patients exposed to AKT (5%), *α*(13%), or pan‐PI3K inhibitors (5%), with none in patients exposed to PI3K inhibitors specific for isoforms other than *α*. Seven of the 491 patients (2%) were admitted for hyperglycemia and all were on PI3k‐*α*inhibitors (Figure 1). The median time from starting treatment to hospital admission was 14 days (range 7–56 days). The average blood glucose value on admission was 538 mg/dl. One of the seven patients was admitted with euglycemic diabetic ketoacidosis (venous blood gas of 7.26, bicarbonate of 13, anion gap of 21, and ketonuria) while being treated with an SGLT2 inhibitor (empagliflozin 10 mg daily) as well as metformin (1000 mg twice daily). The average length of hospital stay was 2.4 days and six of the 7 patients received endocrinology consultation. One patient on a PI3k‐alpha inhibitor discontinued treatment due to hyperglycemia. Among the 26 patients who received copanlisib, the only PI3K/AKT inhibitor administered intravenously in our study, no treatment interruption, dose reduction or hospital admission was observed.

### 3.3 Risk Factors for treatment disruptiond to hyperglycemia

Because treatment‐related hyperglycemia occurred only among recipients of AKT, pan‐PI3k, and PI3K‐alpha targeting inhibitors, analysis of risk factors was restricted to those patients (*n* = 349).

In univariable analysis, baseline HbA_1c_, BMI, glucose, age, and baseline diabetes (defined as either an HbA_1c_ ≥ 6.5% or use of antidiabetic drugs) were significantly associated with increased odds of treatment disruption due to hyperglycemia, while sex was not^25^ (Table 2). A baseline BMI > 25 kg/m^2^ was associated with the largest odds ratio for hyperglycemic events (OR 5.4, 95% CI 2.3–16.0). The highest absolute rate of hyperglycemic events was observed in patients with baseline diabetes (8/23, or 34.7%).

**TABLE 2:** Univariate logistic regression analysis of hyperglycemia development

	Events/Total	Odds ratio	*p*‐value
A1c < 5.7	13/193		
A1c ≥ 5.7	14/55	4.7 (2.1–11.0)	<0.001
BMI < 25	5/134		
BMI ≥ 25	37/214	5.4 (2.3–16.0)	<0.001
Glucose <110	22/262		
Glucose ≥ 110	18/74	3.5 (1.8–7.0)	<0.001
No baseline DM	34/326		
Baseline DM	8/23	4.6 (1.7,11.4)	<0.001
Age < 65	21/230		
Age ≥ 65	21/119	2.1 (1.1–4.1)	0.02
Female sex	34/283		
Male sex	8/66	1.0 (0.4–2.2)	0.98

*Note*: Odds ratio were calculated by Fisher's exact test. The *P* value was considered statistically significant if *p* < 0.05.

Abbreviation: BMI, body mass index.

Baseline HbA_1c_ ≥ 6.5 or use of antidiabetic medication.

When all these covariates were included in a multivariable logistic regression model, the only independently significant predictors were BMI ≥ 25 (4.0, 95% CI 1.3–17.8, *p* = 0.03) and HbA_1c_ ≥ 5.7 (3.4, 95% CI 1.2–9.4, *p* = 0.02). (Table 2).

### 3.4 Use of antidiabetic drugs

Analysis of hyperglycemia management approaches is restricted to recipients of AKT, pan‐PI3k, and PI3K‐alpha targeting inhibitors who were not using antidiabetic drugs at cohort entry (*n* = 331).

**FIGURE 2:**
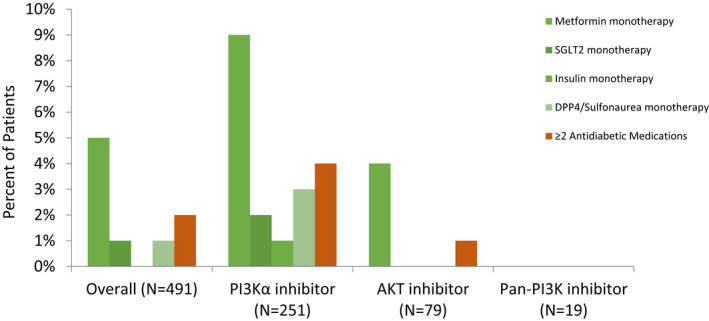
Pharmacotherapy intervention. Antidiabetic medications were initiated to manage hyperglycemia secondary to PI3K/AKT inhibitor treatment are shown. The frequency of starting monotherapy and more than two medications are displayed. AKT, protein kinase B; PI3K, phosphoinositide 3‐kinase

New antidiabetic pharmacologic management was initiated in 62 of 331 patients (18.7%). Thirty‐eight (11.5%) used monotherapy; of these 34 (10.3%) used metformin, one used sulfonylurea and one used DPP‐4 inhibitor. Fourteen patients (37%) used two agents, 3 (8%) used three agents, and 7 (18%) used four agents (Figure 2). Of the 24 patients who received multiple agents, 22 received metformin. Overall, metformin was the most widely used medication (56 users, 16.9%), followed by insulin (14, 4.2%), SGLT2 (12, 3.6%), sulfonylurea (10, 3.0%), DPP4 inhibitor (6, 1.8%), and thiazolidinedione (5, 1.5%) (Figure 2).

We reviewed the timing of when these medications were discontinued. Fifteen of the 56 patients started on metformin (26.7%) discontinued it before discontinuing the PI3K inhibitor. Three of the 13 had no reason documented; four no longer needed it due to improved glycemic control with other interventions; two stopped due to poor efficacy; five stopped due to gastrointestinal side effects, and one stopped due to declining renal function. Of patients started on insulin, 3/14 (21.4%) discontinued before stopping the PI3k inhibitor—in one instance insulin was discontinued at hospital discharge for unclear reasons, and in 2 it was no longer needed due to improved blood glucoses. For new SGLT2 inhibitor users, 3/12 (25.0%) discontinued early; for new sulfonylurea users, 2/10 (20%) discontinued early; for new thiazolidinedione users 1/5 (20%) discontinued early; for new DPP4 users 1/6 (16%) discontinued early. Reasons for discontinuation were not documented for these drugs.

Out of all the subjects using antidiabetic drugs, a single major complication (diabetic ketoacidosis) was noted.

### 3.5 Trends in random glucose measurements

Among patients initiating new antidiabetic drug treatment, random plasma glucose levels were measured during the period of PI3K/AKT inhibitor treatment on a median of seven different days (IQ range 4–10), with a median of one test every 14 days.

Overall, 49.9% of patients (174/349) received AKT, *α‐*, or pan‐PI3K inhibitors experienced Grade 2 or above hyperglycemia. Twenty‐two percent (77/349) and 4% (14/349) of these patients developed hyperglycemia Grade 3 and Grade 4 or higher.

In unadjusted analysis comparing mean glucose in the 30 days before new antidiabetic drug initiation to the mean glucose in the 30 days after, metformin initiation was associated with a decrease of 37 mg/dL (95% CI −59 to −15); insulin use was associated with a decrease of 46 mg/dL (95% CI −123 to 30); SGLT‐2 inhibitor with decrease of 39 mg/dL (95% CI −87 to 9); sulfonylurea with decrease of 15 mg/dL (95% CI −160 to 129); thiazolidinedione with an increase of 13 (95% CI −151 to 178); and DPP4 inhibitor with an increase of 28 mg/dL (95% CI −121 to 177).

Adjusted analysis using multivariable hierarchical linear regression, adjusting for concomitant use of other antidiabetic drugs as well as for age, sex, and baseline glucose found statistically significant reductions in glucose with exposure to metformin [−28 mg/dL (95% CI ‐41 to −16)], SGLT2 inhibitors [−48 mg/dL reduction (95% CI ‐75 to −21)], sulfonylureas [−38 mg/dL reduction (95% CI ‐69 to – 8)], and insulin [−22 (95% CU ‐52 to −2)]. Other antidiabetic medications were not associated with any significant change in random glucose.

### 3.6 Validity of chart review

Inter‐rater reliability (calculated as the percent‐time that two reviewers agreed on a metric) was 96% for presence of hyperglycemia‐related treatment interruption and 100% for presence of PI3K/ AKT inhibitor exposure, based on 80 chart reviews. All diabetes drug exposures that contributed to the time‐varying analysis above were reviewed by two reviewers and any discrepancy was resolved by consensus among the coauthors.

## 4 DISCUSSION

Multiple Pan‐PI3K, isoform‐selective PI3K and AKT inhibitors entered clinical testing in the past 2 decades.^15^ Five have received FDA approval for different disease indications. Hyperglycemia is the most common side effect of selective PI3Kα, AKT and Pan‐PI3K inhibitors and an important challenge in management. This study is the largest study to‐date to assess the frequency, outcomes, and clinical interventions for PI3k/AKT inhibition‐related hyperglycemia in patients on clinical trials and being cared for in routine practice.

Consistent with expectations based on mechanism and on clinical trial data, hyperglycemia and hyperglycemia‐related treatment disruption was common in PI3K‐*α*, AKT and Pan‐PI3K inhibitors but not in other selective PI3k inhibitors. Notably there were no hyperglycemia‐related treatment disruptions at all in patients taking selective PI3K inhibitor for a non‐*α* isoform, whereas in the rest of the cohort 12% experienced hyperglycemia‐related treatment disruptions, including 11% with dose interruptions, 9% with dose reductions, and 2% with hospitalization for hyperglycemia management. However, only one patient discontinued treatment due to hyperglycemia. These data highlighting that this common complication is manageable. Hospital admission could happen as early as 7 days from starting treatment, highlighting the need to recognize, monitor and manage this adverse effect from the first week of treatment.

The Solar‐1 randomized clinical trial suggested age, baseline prediabetes or diabetics to be associated with higher incidence of hyperglycemia in PI3K inhibitor use.^17^, ^26^ In our report, analysis of risk factors for hyperglycemia‐related treatment disruption showed expected associations between hyperglycemic events and elevated baseline glucose, HbA_1c_, and BMI. Multivariable modeling suggests that HbA_1c_ and BMI but not age have independent effects on risk. Given that HbA_1c_ > 5.7 is associated with 25% risk of hyperglycemic events, routine testing of HbA_1c_ prior to initiation of PI3K pathway may be helpful. Providers treating with elevated HbA_1c_ or elevated BMI may wish to consider initiating home glucose monitoring and more aggressive dietary changes prior to treatment.

Over 17% of patients with no use of antidiabetic medications at baseline started at least one such drug after initiating a PI3Kα, AKT and Pan‐PI3K inhibitors and 4.5% patients required multiple agents to manage hyperglycemia. Metformin was by far the most widely utilized medication. While there are both pre‐clinical and anecdotal reasons to doubt that metformin is highly effective in the setting of PI3K/AKT inhibition, it is notable that three‐fourths of patients were able to stay on metformin throughout their treatment and glucose levels did decrease after metformin exposure.^9^, ^27^ Given its excellent safety profile and diverse mechanisms of action, it would be premature to conclude that metformin is not an appropriate first‐line treatment for PI3K/AKI inhibitor induced hyperglycemia.

While metformin remains a management cornerstone, it may not be sufficient. Currently there is no secondary agent that is widely accepted when hyperglycemia remains uncontrolled with metformin. Several anecdotal reports of a robust anti‐glycemic response to SGLT2 inhibitors, along with mechanistic evidence that SGLT2 inhibition may increase the effectiveness of PI3K inhibitors in mice, have led some to suggest that these agents are preferred after or in place of metformin.^9^, ^27^, ^28^ Its efficacy in this setting is being studied in multiple trials.^29^, ^30^, ^31^ Our study provides support for that hypothesis with both crude and adjusted analyses showing greater reductions in blood glucose after SGLT2 inhibitor initiation than any other drug classes. However, the occurrence of one case of euglycemic DKA in this cohort is notable given that only 15 individuals (including 12 new‐users) received SGLT2 inhibitor while on a PI3K/AKT inhibitor. Rare incidence of euglycemia DKA has been reported with SGLT2 inhibitor use.^32^ This experience and the existence of an additional case report in the literature suggest that DKA is rare but significant AE with this combination of therapies.^33^

We identified other potentially effective pharmacologic treatments, notably sulfonylureas or insulin, that relieve hyperglycemia by increasing insulin levels. Substantial pre‐clinical evidence suggests that this approach may undermine the effectiveness of PI3K/AKT inhibitors, as it could be analogous to giving estradiol to women on anti‐estrogen therapy.^34^ SGLT2I inhibitor provide an alternate route for glucose disposal and relieve the need to additional insulin.

These considerations, along with its apparent effectiveness in lowering glucose, make SGLT2 inhibitorsreasonable second line agents after metformin, but patients should be carefully counseled on the signs and symptoms of DKA. We have found that remote monitoring of ketosis using urinary dipsticks or capillary beta‐hydroxybutyrate meters are useful but may lead to complex decision‐making, as patients may have elevated ketones without acidosis and the need for intervention in that scenario is unclear. Current pharmacological interventions that may be considered are summarized in Table 3.

**TABLE 3:** Glucose lowering medications approved by the FDA

Class of drug	Mechanism of action	Major advantages	Major disadvantages
Biguanide (metformin)	Complex; includes inhibition of hepatic blood glucose production	Recommended as first line treatment; excellent overall safety profile, low cost	Effectiveness in setting of PI3K inhibition not well‐established; gastrointestinal side effects (nausea, diarrhea)
Sulfonylureas	Stimulate insulin release	Appears to be effective; few side effects apart from hypoglycemia	Theoretically may undermine effectives of PI3Kinhibitor by activating PI3k through insulin pathway; risk of hypoglycemia
SGLT2 inhibitors	Block reabsorption of glucose from urine	Appears to be particularly effective; mechanism of action is independent of insulin	Increases risk of dehydration, can lower eGFR transiently, possibly significant risk of euglycemic DKA
Thiazolidinediones	Decrease insulin resistance	Acts without raising insulin levels	Some evidence of low effectiveness in setting of PI3K inhibition
DPP‐4 inhibitors	Increase glucose‐dependent pancreatic insulin release Decrease glucagon release	Well‐tolerated with few side effects	Some evidence of low effectiveness in setting of PI3K inhibition
GLP‐1 receptor agonists	Increase pancreatic insulin release Suppress glucagon secretion Suppress appetite	Potent agents with insulin‐independent mechanisms of action	Little experience with use in setting of PI3Kinhibition; significant gastrointestinal side effects
Insulin	Stimulate glucose uptake Inhibit glucose production	Easily titrated, dose can be raised until it is effective	Theoretically may undermine effectiveness of PI3K inhibitor by activating PI3k through insulin pathway; risk of hypoglycemia; often requires extensive patient education

Abbreviations: DKA, diabetic ketoacidosis; eGFR, estimated glomerular filtration rate; PI3K, phosphoinositide 3‐kinases.

Dietary counseling, including potentially use of ketogenic diets, may also play an important role in managing hyperglycemia in the setting of PI3K/AKT inhibition. This study is unable to speak to the role of such dietary modifications as patient diets were not well‐captured in the retrospective data used. During the period of this study, patients were frequently counseled to limit carbohydrate intake both informally and through consultation with registered dieticians, but were not typically advised to attempt to adopt a ketogenic diet.

This manuscript has limitations. The only option for determining key drug exposures and outcomes was retrospective chart review, which could not be blinded. Treatment doses in patients on clinical trials might be higher or lower than used in routine practice, perhaps affecting adverse event rates. But, for the main drivers of study conclusions—which drugs a patient was exposed to, and occurrence of major treatment disruption due to hyperglycemia—documentation was unambiguous and inter‐rater reliability was high. Due to the limitations of randomly collected glucose data, conclusions about the effectiveness of different antidiabetic drugs should be interpreted cautiously and deserve validation with prospective data collection that addresses these limitations.

Despite these limitations, this manuscript provides an overview of an area of rapidly evolving clinical practice, moving guidelines from purely anecdotal and mechanistic basis to systematically collected and analyzed data. It shows that significant hyperglycemia occurs in a substantial proportion of patients receiving PI3K or AKT inhibitors; that it is particularly likely in patients with baseline diabetes, pre‐diabetes, or elevated BMI that it is manageable with dose interruption, dose modification and/or pharmacologic management; and that SGLT2 inhibitors are a promising second‐line option after metformin, provided that all steps are taken to mitigate the potential risk of euglycemic D.

## CONFLICT OF INTEREST

JJH has received research funding from Boehringer Ingelheim, Bristol‐Myers Squibb, CytomX, Debiopharm, Eli Lilly, Genoscience, Novartis, Pfizer, Polaris, Loxo Oncology, Yiviva, Zymework, and personal fees from Adaptimmune, CytomX, Eisai, Eli Lilly, Exelexis, Merck, QED, Research to Practice, MORE Health, HCC Connect.

## DISCLOSURES

DL received consulting fees from Pfizer, Invitae and Heron Therapeutics. AD received honoraria from: Ignyta/Genentech/Roche, Loxo/Bayer/Lilly, Takeda/Ariad/Millenium, TP Therapeutics, AstraZeneca, Pfizer, Blueprint Medicines, Helsinn, Beigene, BergenBio, Hengrui Therapeutics, Exelixis, Tyra Biosciences, Verastem, MORE Health, Abbvie, 14ner/Elevation Oncology, Remedica Ltd., ArcherDX, Monopteros, Novartis, EMD Serono, Melendi, Liberum, Repare RX, Chugai, Merus, Chugai Pharmaceutical, Nuvalent, mBrace, AXIS, EPG Health, Harborside Nexus, Liberum, RV More, Ology. Associated research paid to insitution: Pfizer, Exelixis, GlaxoSmithKlein, Teva, Taiho, PharmaMar. Royalties: Wolters Kluwer; OTHER: Merck, Puma, Merus, Boehringer Ingelheim. CME honoraria: Medscape, OncLive, PeerVoice, Physicians Education Resources, Targeted Oncology, Research to Practice, Axis,Peerview Institute, Paradigm Medical Communications, WebMD, MJH Life Sciences, AXIS, EPG Health, JNCC/Harborside. MDG received consulting fees from Novartis, Pfizer, and Scorpion Therapeutics; he is an inventor on a patent (pending) for Combination Therapy for PI3K‐associated Disease or Disorder; and he is a co‐founder, shareholder, and consultant of Faeth Therapeutics. SH acted as an advisory board member for ADC Therapeutics America, Inc. KJ has served as a consultant for Novartis, Genentech, Lilly Pharmaceuticals, Taiho Oncology, Jounce Therapeutics, Astra Zeneca, Spectrum Pharmaceuticals, ADC Therapeutics, Pfizer, BMS, AbbVie, Seattle Genetics, Blueprint Medicines, Biotheranostics and has received research funding (to the institution) from Novartis, Pfizer, Clovis Oncology, Genentech, Astra Zeneca, ADC Therapeutics, Novita Pharmaceuticals, Debio Pharmaceuticals, Puma Biotechnology, Zymeworks, Immunomedics, Merck/VelosBio. JHF has consulted for Boehringer Ingelheim, Janssen Pharmaceuticals, and Genentech. All other authors have no relevant disclosures to report.

## Data Availability

The data that support the findings of this study are available on request from the corresponding author. The data are not publicly available due to privacy or ethical restrictions.
